# PHGD: An integrative and user‐friendly database for plant hormone‐related genes

**DOI:** 10.1002/imt2.164

**Published:** 2024-01-08

**Authors:** Shuyan Feng, Zhuo Liu, Huilong Chen, Nan Li, Tong Yu, Rong Zhou, Fulei Nie, Di Guo, Xiao Ma, Xiaoming Song

**Affiliations:** ^1^ School of Life Sciences/Library North China University of Science and Technology Tangshan Hebei China; ^2^ College of Grassland Science and Technology China Agricultural University Beijing China; ^3^ Department of Food Science Aarhus University Aarhus Denmark; ^4^ College of Horticultural Science & Technology, Hebei Normal University of Science & Technology Qinhuangdao Hebei China

## Abstract

Plant Hormone Gene Database (PHGD) database platform construction pipeline. First, we collected all reported hormone‐related genes in the model plant Arabidopsis thaliana, and combined with the existing experimental background, mapped the hormone–gene interaction network to provide a blueprint. Next, we collected 469 high‐quality plant genomes. Then, bioinformatics was used to identify hormone‐related genes in these plants. Finally, these genetic data were programmed to be stored in a database and a platform website PHGD was built. PHGD was divided into eight modules, namely Home, Browse, Search, Resources, Download, Tools, Help, and Contact. We provided data resources and platform services to facilitate the study of plant hormones.

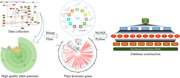

Plant hormones are small amounts of organic compounds produced by plants, which can promote or inhibit diverse physiological processes [1, 2, 3]. Plant hormones mainly include auxin (AUX), ethylene (ETH), cytokinin (CK), abscisic acid (ABA), gibberellin (GA), brassinosteroids (BRs), jasmonic acid (JA), strigolactones (SLs), salicylic acid (SA), melatonin (MT), and polypeptide (PEP) hormones [4, 5, 6]. Each kind of plant hormone plays its role in the environmental response, defense response, plant growth, and development [7]. For example, CKs have been implicated in cell division, root and bud development, senescence, and chloroplast development [8]. Polypeptide hormones play important roles in plant growth, development, and adaptation to the environment, including precursor of peptide (PROPEP) [9, 10], phytosulfokine‐α (PSK) [11, 12], Ralf‐like (RALF) [13], epidermal patterning factor/epidermal patterning factor‐like (EPF/EPFL) [14], clavata3/ESR‐related (CLE) [15], and root growth factor (RGF) [16]. MT plays an important role in different aspects of plants, such as salt and drought stresses [17, 18]. Moreover, they can interact with each other to form a huge network to regulate various biological processes [19, 20]. For instance, MT has crosstalk with SA, JA, AUX, and ABA, which further contributed to plant disease resistance [21].

Plant hormone‐related genes are involved in a variety of pathway mechanisms, including biosynthesis, metabolism, perception, signal transduction, and transport pathways [22, 23]. Therefore, it is very difficult to comprehensively analyze the complex interaction network and action mechanism of plant hormones. Much work has been done to contribute to exploring the mechanism of some plant hormones. For example, four main auxin signaling pathways have been reported, that is, transport inhibitor response 1/auxin signaling F‐boxes (TIR1/AFBs)‐auxin/indole‐3‐acetic acid (AUX/IAA)‐auxin response factors (ARFs) [24], transmembrane kinase 1 (TMK1)‐IAA32/34‐ARFs [25], TMK1/auxin‐binding protein1 (TMK1/ABP1)‐rho‐related protein from plants 2/6 (ROP2/6)‐pin‐formeds (PINs) [26], and S‐phase kinase‐associated protein 2a (SKP2A)‐*Arabidopsis thaliana* homolog of e2f c/ATDPB (E2FC/DPB) [27]. Nevertheless, the last signaling pathway has been proposed for many years, but it is highly controversial and requires more evidence [27]. In addition, four enzymes in the melatonin biosynthesis pathway have been reported, including tryptophan decarboxylase (TDC), tryptamine 5‐hydroxylase (T5H), serotonin *N*‐acetyltransferase (SNAT), and acetyl serotonin methyl transferase (ASMT) [28, 29]. However, the mechanism of melatonin signal transduction pathway is still not clear. Moreover, the mechanism of polypeptide hormone pathway also needs to be explored.

The study of plant hormone‐related pathways and mechanisms of action requires lots of human and experimental data verification, so it is a very large and time‐consuming work. Another effective approach is through big data and resource integration analysis. At present, only a few related databases have been released about plant hormones. Among them, the GSHR database (Gene Set‐Level Analyses of Hormone Responses in *Arabidopsis*, http://bioinfo.cemps.ac.cn/GSHR/) is only for information integration of hormone genes in *Arabidopsis thaliana* [30]. The other one plant hormone data platform (https://plant-hormones.info/) is currently unavailable. As far as we know, to date, there is no database showing the distribution of 11 plant hormones in large‐scale different plant kingdoms, involving biosynthesis, metabolism, perception, signal transduction, and transport pathways. Therefore, to promote the comprehensive study of plant hormone pathways and mechanisms, we described a comprehensive complex interaction network of plant hormones, using bioinformatics methods to search for hormone‐related genes in 469 high‐quality plant genomes. Based on the deletion of genes in lower plants, the origin of each hormone pathway was explored. Finally, a data‐sharing platform was established to help researchers study plant hormone genes.

## RESULTS

### Collection of *Arabidopsis* hormone genes and genomic data of 469 species

All *A. thaliana* hormone‐related genes were manually screened from the literature and the TAIR database (https://www.arabidopsis.org/index.jsp). Initially, a comprehensive search was performed on PubMed, Web of Science, and Google Scholar using keywords such as “AUX,” “ABA,” “plant hormone,” “phytohormone,” “*Arabidopsis*,” and “phytohormone pathway.” The retrieved publications were then preliminarily checked to eliminate false‐positive papers. For phytohormone pathway information, the Kyoto Encyclopedia of Genes and Genomes database for phytohormone pathway information was consulted in addition to the literature. Finally, we collected and sorted out 914 plant hormone genes of the model plant *A. thaliana* from 973 publications, including crucial genes in biosynthesis, metabolism, perception, signal transduction, and transport pathways (Supporting Information S1: Tables S1 and S2). After a serial genome data collection and filtering, 469 species with high‐quality gene annotation were used in this study, including 314 dicots, 85 monocots, six magnoliids, three basal angiosperms, eight gymnosperms, six ferns, eight bryophytes, and 39 algae (one glaucophyte, one prasinodermophyte new proposed in 2020 [31], eight red algae, 22 green algae, and seven charophytes) (Supporting Information S1: Tables S3 and S4). At the same time, these species can also be divided into flowering (408) and nonflowering (61) species. The gff files, coding sequences (CDSs), and protein sequences of 469 examined species were collected and downloaded according to the information of Plant Genomes (https://www.plabipd.de/plant_genomes_pn.ep), which was further used for identifying the plant hormone genes.

### Construction of cross‐network among plant hormone genes

**Figure 1:**
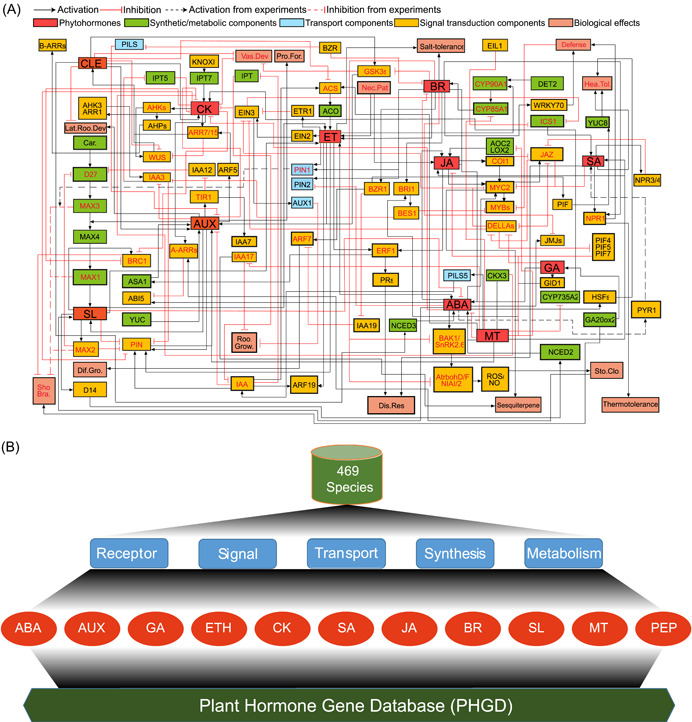
Topology of 11 phytohormones networks in *Arabidopsis* and architecture of Plant Hormone Gene Database (PHGD). (A) Topology of 11 phytohormones networks in *Arabidopsis*. The larger and red‐filled boxes represent plant hormones. There are 104 nodes in the network topology. Among these, 49 crucial nodes, which are red or filled with red, have three lines connected. The color and line code are explained in the picture. (B) The architecture of PHGD. It consists of eight modules, home, browse, search, resources, download, tools, help, and contact. Moreover, it covers 469 species and contains a comprehensive list of 11‐type plant hormone genes involved in receptor, signal, transport, synthesis, and metabolism. Dif.Gro, Differential Growth; Dis.Res, Disease Resistance; Hea.Tol, Heat Tolerance; Lat.Roo.Dev, Lateral Root Development; Nec.Pat, Necrotrophs Pathogens; Pro.For, Procambium Formation; Roo.Grow, Root Growth; Sho.Bra, Shoot Branching; Sto.Clo, Stomatal Closure; Vas.Dev, Vascular Development.

We collected and sorted out the existing literature related to plant hormone gene crosstalk and constructed a network of 11‐type plant hormone genes in *Arabidopsis*, which involved components of biosynthesis, metabolism, perception, transduction, and transport pathways (Figure 1A). Furthermore, multiple biological effects, such as disease defense, salt stress, heat stress, stomatal closure, branching, and vascular development, were also included. A total of 104 nodes were found in the crosstalk network. Among these, 49 crucial nodes with three lines connected play an infinitely important role in hormone interactions. The construction of a complex regulatory network enabled us to understand the signal crosstalk mechanism more systematically.

### Identification of plant hormone genes

Combined with the references, the crucial components of each plant hormone pathway were selected in *A. thaliana*. For example, lonely guy (LOG) and isopentenyltransferase (IPT) are key rate‐limiting enzymes in the cytokinin synthesis pathway, and PURINE PERMEASE (PUP), equilibrative nucleoside transporter (ENT), and ATP‐binding cassette g14 (ABCG14) transport proteins that play important roles in the cytokinin transport pathway. We also summarized the key synthetic components of melatonin, a new phytohormone member that has been added in recent years, and the PMTR1 receptor. In addition, we have added the peptide hormones PROPEP, PSK, RALF, and EPF/EPFL, which have not been overexplored. Then, we collected domain information of Pfam and protein sequences of these crucial components for a blast to identify these components in the 469 species by Pfam database (version 34.0) [32] and Blast (version 2.10.1). The Pfam database was used to identify all members of each family according to the previous reports [32, 33, 34], and blastp was used to obtain the similarity relation of hormone component proteins (*E* value < 1e^−5^). For blast results, we set filter standards of hormone components for higher and lower plants, respectively. For higher plants, the main reason for setting filtering criteria for different components is the multiple relationships of the reference genome. For example, *A. thaliana* and Chinese cabbage are theoretically three times as large [35]. However, due to the large amount of gene loss, the number of identified hormone genes in Chinese cabbage should be about 1.2 to 2 times that of *A. thaliana*. Based on this theory, we synthesized the preliminary identification results of all species to determine an optimal threshold [36, 37, 38] and the threshold value was used as the filtering standard for the hormone components. For lower plants, the filter standards were used according to the previous report, such as *Chara braunii* and *Physcomitrella patens* [39]. We selected 104 important components of each pathway of 11 plant hormones and collected their domain information and protein sequences for blast with related criteria (Supporting Information S1: Tables S5–7). We sorted out and obtained the gene numbers of 104 pathway components in all plant taxa we studied (Supporting Information S1: Tables S8–19).

### Exploring the origin of each pathway of 11 plant hormones

We selected 64 representative species in each plant taxon to display the distribution of components in each pathway of 11 plant hormones (Supporting Information S1: Table S20) and determined the origin node of the hormone pathway based on the presence and deletion of genes. First, we counted the distribution of 11‐type hormone pathway components in 469 species (408 higher plants and 61 lower plants) (Supporting Information S1: Table S19). Then, on the premise of avoiding species loss, we selected 64 representative species (11 higher plants and 53 lower plants) of different taxa from lower algae to higher dicots, counting the presence of each pathway component in these species. If this pathway component is present in the species, we define it as “1,” and if it is not, we define it as “0” (Supporting Information S1: Table S20). Finally, we inferred the origin nodes of different paths according to this statistical result. Here, we selected AUX, CK, PEP hormones, and MT as examples.

#### AUX

The homologous proteins of AUX synthetic component YUCCA (YUC) are distributed in all plant taxa and tryptophan biosynthesis proteins) are only present in land plants. The results suggest that the auxin synthesis pathway originated from the common ancestor of land plants. The AUX receptor TIR1/AFB was found in all plant taxa and ABP1 was found in three green algae (*Chlorella variabilis, Micractinium conductrix*, and *Chlamydomonas reinhardtii*), five charophytes (*Klebsormidium nitens, C. braunii, Mesotaenium endlicherianum, Penium margaritaceum*, and *Spirogloea muscicola*), and almost all land plants. The results suggest that the complete AUX perception pathway originated in green algae. The signal transduction component AUX/IAAs, small auxin‐upregulated RNA, and ARF exist in charophytes and land plants. Other critical signal transduction components ROP, Topless, and TMK were found in all plant taxa. DPB, E2FC, and SKP2A are highly scattered among the species we studied and only simultaneously exist in 63 higher plants (Supporting Information S1: Table S19). Due to the high controversy of the SKP2A/E2FC/DPB pathway, we only explored the distribution of DPB, E2FC, and SKP2A and they are not to be a reference for the origin study. In short, our results indicate that AUX signal transduction originated in charophytes. The transport components AUXIN1 (AUX1)/like AUX1, PIN‐formed (PIN)/PIN‐likes, and ABC transport families exist in all plant taxa, suggesting that the AUX transport pathway originated from glaucophytes. The metabolic components GRETCHEN HAGEN 3 were first found in the green algae *C. variabilis*, followed by charophytes *K. nitens* and *M. endlicherianum*, as well as all land plants. These results indicate that the AUX metabolic pathway originated in green algae.

#### CK

CK biosynthetic components LOG and IPT are present in all plant taxa, which indicates that the CK biosynthetic pathway originated in glaucophytes. The metabolic component cytokinin oxidase only exists in land plants, indicating that the CK metabolic pathway originated from the last common ancestor of land plants. CK receptor *Arabidopsis* histidine kinase was only found in charophytes and terrestrial plants, which indicates that the CK perception pathway originated from charophytes. The signal transduction components *Arabidopsis* histidine‐containing phosphotransmitter (AHP) and cytokinin response factors exist in all plant taxa except for red algae. In addition, type‐A *Arabidopsis* response regulators (ARRs) are absent only in some red algae, and type‐B ARRs are absent in glaucophytes, all red algae, some green algae, and some charophytes. These signal transduction components simultaneously exist in prasinodermophytes, some green algae and charophytes, and terrestrial plants, indicating that CK signal transduction may originate from prasinodermophytes. The transport component PUP was only found in two red algae (*Cyanidiococcus yangmingshanensis* and *Cyanidioschyzon merolae*), three green algae (*Asterochloris glomerata, C. variabilis*, and *Chromochloris zofingiensis*), and land plants. Other transport components ENT and ABCG14 were found in each taxon. The results imply that the transport pathway originated in red algae.

#### PEP hormones

The PROPEP family is present in eight chlorophytes (*A. glomerata, Coccomyxa subellipsoidea, C. variabilis, Chlamydomonas incerta, Chlamydomonas eustigma, Edaphochlamys debaryana, Gonium pectorale*, and *C. zofingiensis*), five charophytes (*Chlorokybus atmophyticus, K. nitens, M. endlicherianum, P. margaritaceum*, and *S. muscicola*), and land plants, which indicates that it originated in chlorophytes. Furthermore, we found that the PSK family only exists in gymnosperms and angiosperms, indicating its origin from the last common ancestor of gymnosperms. RALF and EPF/EPFL polypeptide hormone families exist in all land plants but not in algae, which suggests that they both originated from the last common ancestor of land plants.

#### MT

In our study, we found that the melatonin synthetic component TDC first had its homologous protein in green algae, SNAT in glaucophytes, and ASMT in prasinodermophytes. These three synthase homologs are simultaneously present in three green algae (*C. subellipsoidea, C. zofingiensis*, and *Monoraphidium neglectum*), one chlorophyte (*M. endlicherianum*), and most land plants. We did not find the T5H gene in *A. thaliana* as a search sequence for blast, so we did not study it. According to the results of TDC, SNAT, and ASMT, we speculated that the melatonin synthetic pathway originated in green algae. The homologous protein of the receptor PMTR1 was found in green algae, charophytes, and land plants. Therefore, the melatonin perception pathway also originated in green algae.

### PHGD construction

**Figure 2:**
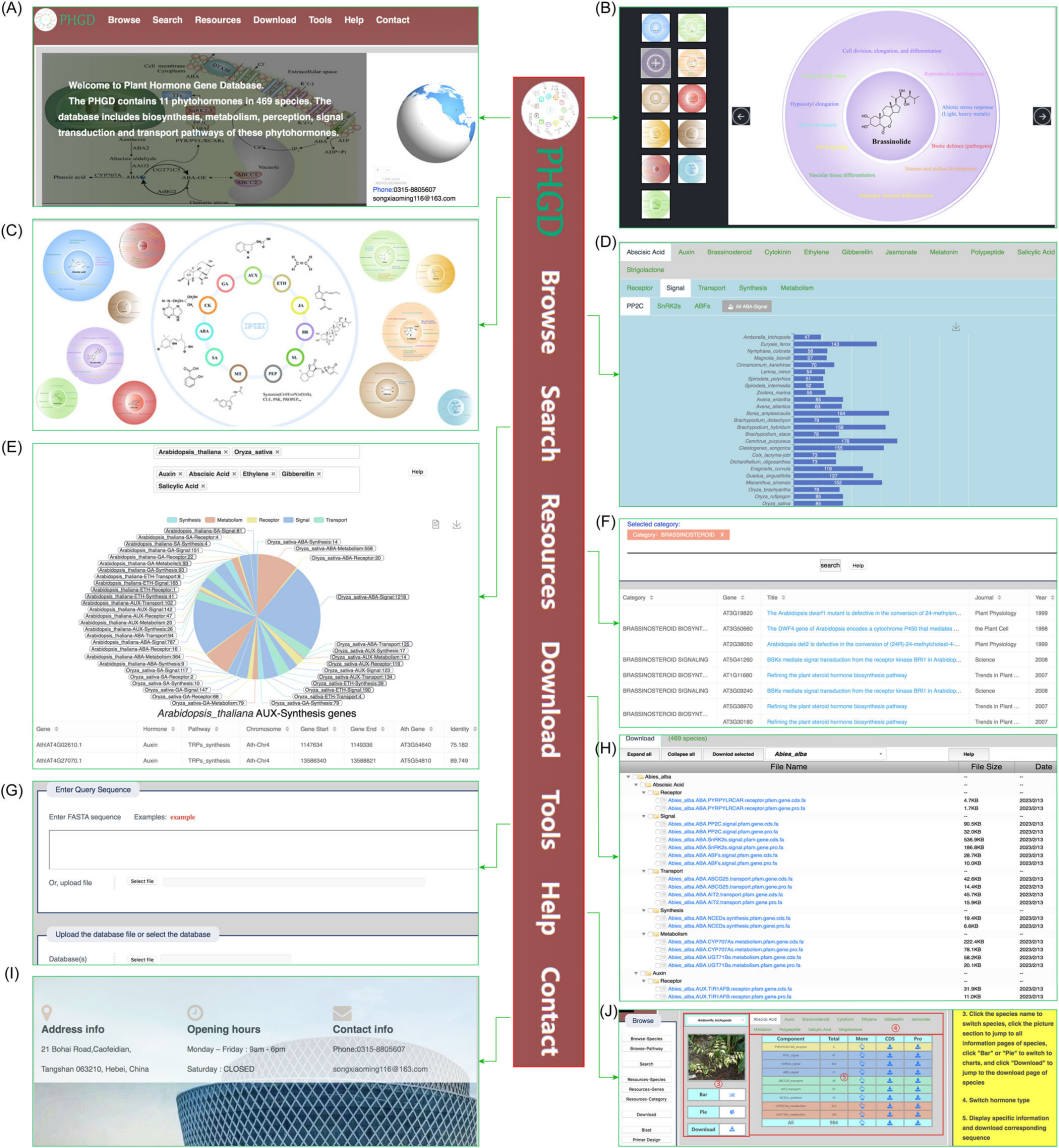
Overviews of main interfaces of the Plant Hormone Gene Database. (A–C) Home, (D) Browse, (E) Search, (F) Resources, (G) Tools, (H) Download, (I) Contact, and (J) Help.

By summarizing the above‐identified hormone genes, the comprehensive PHGD (http://phgd.bio2db.com/) was constructed with a user‐friendly web interface referencing the previous reports [40, 41, 42]. PHGD includes 11 types of plant hormones from 469 species (Figure 1B). Moreover, PHGD contains 104 components of synthesis, metabolism, receptor, signal, and transport pathways of each plant hormone (Figure 1B). In addition, PHGD integrates the functions and interaction networks of 11 plant hormones (Figure 2A–C). Here, we present an overview of PHGD interfaces, including Browse, Search, Resources, Download, Tools, Help, and Contact interfaces, to help users easily use our database (Figure 2A).

#### Browse

We provide two modes “Species” and “Pathway” for users to browse plant hormone genes (Figure 2D). In “Species” mode, we have classified species, and users can easily locate their species according to the classification of queried species. Then, users can view all components in the pathways of each plant hormone in a table. The “MORE” column in the table contains specific gene information, such as gene name, Pfam or blast information, CDSs, and protein sequences. The sequences can also be downloaded directly in the “CDS” and “Pro” columns of the table. By clicking “Bar” or “Pie,” users can see the number or ratio of each hormone pathway. Moreover, users can download the plant hormone data of each species according to their needs via clicking “Download.” In “Pathway” mode, users can view plant hormone genes of all species of each component in one pathway, and we display the number of genes in each species through a bar chart. Users can download data for all components in the hormone pathway.

#### Search

Users could search the related genes of 11 plant hormones in each species (Figure 2E). The number and ratio of genes in each pathway are displayed in a pie chart. Users can also see the data in the data view and use it directly for research. Search results contain rich information, including gene name, Pfam (pfam id, domain, *E* value, and family) or blast (chromosome, start, end, homologous genes with *A. thaliana*, and annotation), CDSs, and protein sequences.

#### Resources

In the resource section, species, genes, and category information are included (Figure 2F). We provide the Latin name, taxonomy, genomic information, published articles, and data download links of the species used in this study. In addition, we also provide details of *Arabidopsis* plant hormone genes, including gene name, pathway, references, journal, publication year, and authors. Users can directly download this information for related research.

#### Tools

We provide two popular tools, Blast and Primer Design, to help users perform plant hormone gene analysis (Figure 2G). The Blast tool was used to help users conduct CDS or protein sequence alignment. We constructed a user‐friendly interface and built a Blast database using plant hormone genes of each pathway from 469 species. All users can easily conduct sequence alignment by copying sequences to the frame or uploading sequences in Fasta format. We also developed a Primer Design tool to help users design primers for plant hormone genes or other assigned genes.

#### Download, contact, and help

Users can obtain sequences of plant hormone genes from the Download interface on demands for research (Figure 2H). In addition, we provide the address, email, and phone number for users to contact us for any related questions (Figure 2I). In the help section, a detailed manual and a video are provided for users to know how to use each interface of the PHGD database (Figure 2J). We also encourage users to submit the new plant hormone genes to us to further enrich the database.

### Examples of the use of PHGD

**Figure 3:**
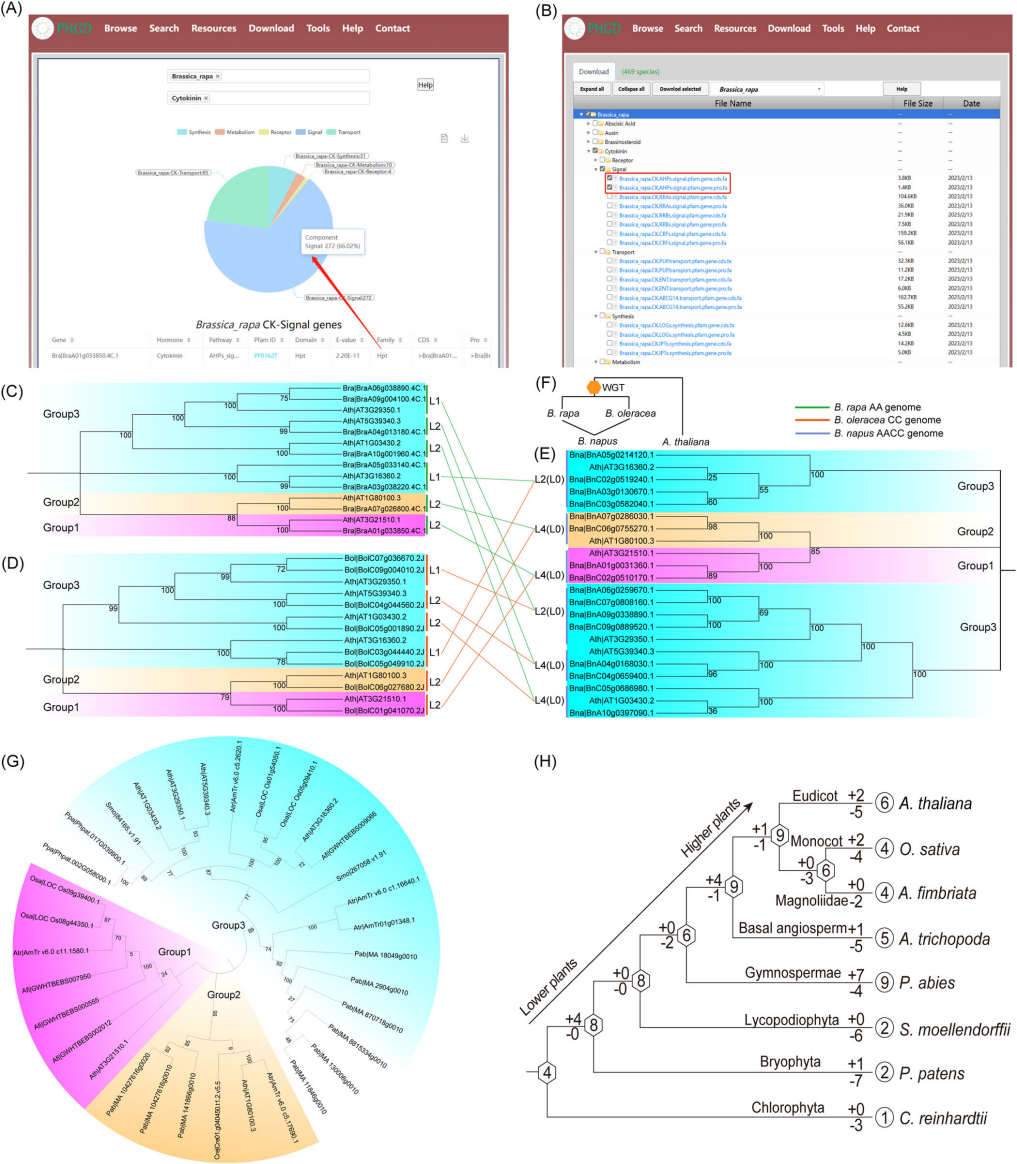
An application demonstration of Plant Hormone Gene Database data analysis. (A) Data query. (B) Data download. (C–F) With *Arabidopsis* as a reference, the expansion analysis of the *Arabidopsis* histidine‐containing phosphotransmitter (AHP) family of Chinese cabbage, *Brassica oleracea* and *Brassica napus*. Panel (C) represents *Arabidopsis* and Chinese cabbage. Panel (D) represents *Arabidopsis* and *B. oleracea*. (E) represents *Arabidopsis* and *B. napus*. Panel (F) represents the evolutionary relationship of four studied species. “L” and “D” represent loss and duplication, respectively. The numbers after “L” and “D” represent the number of genes. (G) The maximum‐likelihood tree of AHP family genes from eight representative species. (H) Schematic diagram of gain and loss of AHP gene family in plants. The numbers in the hexagons and circles represent the number of AHP genes in ancestors and existing species, and the + and − signs represent the gain and loss of genes, respectively. WGT, whole‐genome triplication.

We selected as examples the download and evolutionary analysis of the *AHP* gene family data belonging to the CK signal transduction pathway in plants such as Chinese cabbage. First, search the target species and pathway from the search page, view the relevant information, and identify the target gene of interest (*AHP* gene family) (Figure 3A). We then selected the sequence file of the target species' *AHP* gene family from the Download page (Figure 3B). After the verification of Pfam (version 34.0) [32], SMART (version 9, https://smart.embl.de) [43], and CDD databases (version 3.17, https://www.ncbi.nlm.nih.gov/Structure/cdd/cdd.shtml) [44], we identified six *A. thaliana AHP* family members, consistent with the previous report [45]. Next, the neighbor‐joining method was used for sequence alignment and the phylogenetic tree was constructed using MEGA X software [46]. Then, according to our previously published method [47, 48, 49], the expansion analysis of *AHP* gene family of Chinese cabbage, *Brassica oleracea*, and *Brassica napus* was carried out using *A. thaliana AHP* gene family as reference (Figure 3C–E). Based on the multiploidy relationships that species experienced (Figure 3F), in theory, the relationship between the number of genes of *A. thaliana*, Chinese cabbage, *B. oleracea*, and *B. napus* is 1:3:3:6 [49]. The results showed that the homologous gene corresponding to each *A. thaliana AHP* gene was lost. The number of *A. thaliana*, Chinese cabbage, *B. oleracea*, and *B. napus AHP* genes downloaded from PHGD were six, eight, eight, and 16, respectively. *B. napus* is a hybrid species of Chinese cabbage and *B. oleracea*, so the sum of the number of *AHP* genes of Chinese cabbage and *B. oleracea* is equal to the number of *AHP* genes of *B. napus*. In combination with phylogenetic trees, we found that the expansion of *AHP* family in *B. napus* was indirect expansion caused by hybridization, while that in Chinese cabbage and *B. oleracea* was direct expansion caused by genome duplication.

To further explore the evolutionary trajectory of *AHP* genes, the *AHP* sequences of eight representative species (*A. thaliana, Oryza sativa, Aristolochia fimbriata, Amborella trichopoda, Picea abies, Selaginella moellendorffii, P. patens*, and *C. reinhardtii*) in plant kingdom were downloaded from our PHGD platform. Then, the Mafft (version 7.475) was used for sequence alignment [50] and FastTree (version 2.1) was used to construct the phylogenetic tree (Figure 3G) [51]. Finally, Notung software (version 2.9.1.5) was used to analyze the duplication and loss of gene families [52]. The family size of the ancestral node was inferred according to the previous method [53] (Figure 3H). The results show that the ancestors of land plants and *C. reinhardtii* contained four *AHP* genes. The loss is more serious in lower plants. Among them, three *AHP* genes of *C. reinhardtii* have been lost and zero *AHP* genes have been obtained. Seven *AHP* genes of *P. patens* and six *AHP* genes of *S. moellendorfii* have been lost, and one *AHP* gene and zero *AHP* gene have been obtained, respectively. The common ancestor of land plants contains eight *AHP* genes and the common ancestor of seed plants contains six *AHP* genes. The common ancestor of angiosperm plants contains nine *AHP* genes. The common ancestor of eudicot plants and monocot plants contains nine *AHP* genes. From the number of the *AHP* family members in lower plants to higher plants, it exhibits an expansion and evolutionary pattern.

## DISCUSSION

In this study, through extensive literature and investigations, we identified 914 genes involved in 11 plant hormone pathways of the model plant *A. thaliana*, and then integrated lots of previous studies to map the complex network of 11 plant hormone interactions. The plant hormone pathway in this network contains 104 components, involving biosynthesis, metabolism, perception, signal transduction, and transport pathways. Among these, 49 nodes are closely related to at least three components, providing the core nodes for the study of plant hormone interactions and an overall blueprint for hormone regulation of plant growth and development. Then, through homologous searches, we identified genes that are key components of 11‐type plant hormone pathways in 469 plants (408 flowering plants and 61 nonflowering plants). Then, based on these big data, the origin of plant hormone genes in each pathway was explored. Finally, a user‐friendly plant hormone database platform was constructed to assist people in the study of plant hormones. We selected data analysis of a gene family to indicate one use of our PHGD, while the effect of PHGD is far from this example.

Currently, as far as we know, the only available plant hormone website is GSHR, a website dedicated to providing a resource service for *A. thaliana* plant hormones, which has already contributed greatly to hormone research. Compared with PHGD, GSHR only mainly focuses on *A. thaliana*, which severely limits the study of hormones in other plants. However, our PHGD database contains most of the representative species whose genomes have been sequenced so far, which can provide rich data resources for studying the regulatory mechanisms of hormone‐related genes in more plants. In addition, GSHR is not comprehensive enough in terms of hormone types. Moreover, GSHR has not been updated since 2018, and the detailed hormone pathways of most species are lacking in GSHR. Although PHGD makes up for the above deficiencies, some hormone members are only identified by bioinformatics methods and lack experimental functional validation, which provides rich gene resources for plant hormone‐related researchers and breeders. Researchers can conduct in‐depth functional verification of specific genes according to their needs.

Overall, this is the first large‐scale collection and integration of comprehensive plant hormone pathways. As an important platform of plant hormone data resources, PHGD will greatly promote the related research of plant hormone genes. In the future, with the release of more and more genomes and innovation in hormone research, we will also continue to improve and update this database.

## CONCLUSION

In conclusion, we summarized and mapped the complex interaction network of plant hormones to provide a blueprint for hormone research. Through comprehensively identifying hormone genes from biosynthesis, metabolism, perception, signal transduction, and transport pathways in 469 plant genomes, we constructed the first comprehensive plant hormone gene resource platform. In the future, we will continuously improve and update the plant hormone genes of newly assembled genomes in our database. We believe this database to become a key resource for the study of plant hormone genes for all related researchers in the world.

## AUTHOR CONTRIBUTIONS

Xiaoming Song conceived the project and was responsible for the project initiation. Xiaoming Song, Shuyan Feng, Zhuo Liu, and Xiao Ma supervised and managed the project and research. The data collection and bioinformatic analyses were led by Xiaoming Song, Shuyan Feng, Huilong Chen, and Xiao Ma. The database construction was led by Xiaoming Song, Zhuo Liu, Tong Yu, Xiao Ma, and Fulei Nie. The manuscript was organized, written, and revised by Xiaoming Song, Shuyan Feng, Xiao Ma, Nan Li, Di Guo. and Rong Zhou. All authors read and approved the manuscript.

## CONFLICT OF INTEREST STATEMENT

The authors declare no conflict of interest.

## Supporting information


**Table S1:** The publication and data sources of all collected hormone genes in *A. thaliana*.
**Table S2:** The description information of all collected hormone genes in *A. thaliana*.
**Table S3:** The classification, genomic data source, and publication information of 469 species.
**Table S4:** The number of 469 species in different taxa used in this study.
**Table S5:** The information of protein structural domain of plant hormone pathway components.
**Table S6:** The genes in *A. thaliana* used for blast plant hormone pathway components to display.
**Table S7:** The standards of blast filter for hormone pathways.
**Table S8:** The number of ABA genes of different pathways through pfam identification.
**Table S9:** The number of AUX genes of different pathways through blast filter and pfam identification.
**Table S10:** The number of BR genes of different pathways through blast filter and pfam identification.
**Table S11:** The number of CK genes of different pathways through pfam identification.
**Table S12:** The number of ETH genes of different pathways through blast filter and pfam identification.
**Table S13:** The number of GA genes of different pathways through pfam identification.
**Table S14:** The number of JA genes of different pathways through blast filter and pfam identification.
**Table S15:** The number of SA genes of different pathways through blast filter and pfam identification.
**Table S16:** The number of SL genes of different pathways through blast filter and pfam identification.
**Table S17:** The number of MT genes of different pathways through blast filter.
**Table S18:** The number of POLYPEPTIDE genes through pfam identification.
**Table S19:** The numbers of hormone components in different pathways.
**Table S20:** The data of heatmap of plant hormone pathway components in 64 representative species.

## Data Availability

All related data sets in this study are available in PHGD (http://phgd.bio2db.com/). Supplementary materials (figures, tables, scripts, graphical abstract, slides, videos, Chinese translated version and update materials) may be found in the online DOI or iMeta Science http://www.imeta.science/.
